# Observational cross-sectional study of *Trichomonas tenax* in patients with periodontal disease attending a Chilean university dental clinic

**DOI:** 10.1186/s12903-019-0885-3

**Published:** 2019-09-04

**Authors:** Casandra Bracamonte-Wolf, Patricio R. Orrego, Christian Muñoz, Daniel Herrera, Joel Bravo, Jorge Gonzalez, Héctor Varela, Alejandro Catalán, Jorge E. Araya

**Affiliations:** 1 Laboratory of Molecular Parasitology, Department of Medical Technology, Faculty of Health Sciences, University of Antofagasta, Angamos Avenue 601, P.O. Box 170, Antofagasta, Chile; 2 Biomedical Departmen, Faculty of Health Sciences, University of Antofagasta, Angamos Avenue 601, P.O. Box 170, Antofagasta, Chile; 3 Department of Dentistry, Faculty of Medicine and Dentistry, University of Antofagasta, Angamos Avenue 601, P.O. Box 170, Antofagasta, Chile; 4 Department of Mathematics, Faculty of Basic Sciences, Universidad de Antofagasta, Angamos Avenue 601, P.O. Box 170, Antofagasta, Chile

**Keywords:** *Trichomonas tenax*, PCR, Periodontal disease, Periodontitis, Gingivitis

## Abstract

**Background:**

The oral flagellated protozoan *Trichomonas tenax* has been associated with patients with periodontal disease. However, no recent studies have been conducted on the prevalence of *T. tenax* in Chile. The aim of this study was to determine the presence of *T. tenax* in patients with periodontal disease, admitted to the Dental Clinic of the University of Antofagasta, Chile, through Polymerase Chain Reaction (PCR) amplification of the beta-tubulin gene.

**Methods:**

An observational, cross-sectional study was conducted on 50 patients diagnosed with periodontal disease, 20 of them with gingivitis and 30 with periodontitis. *T. tenax* was identified by PCR amplification of the beta-tubulin gene. Associations between the protozoan and periodontal disease or the presence of risk factors to establish *T. tenax* infection were determined using the chi-square test and binary logistic regression analysis.

**Results:**

*T. tenax* was present in 28 out of 50 (56%) of patients with periodontal disease, and was more prevalent when associated with periodontitis (21 out of 30; 70%) than dental plaque-induced gingivitis (7 out of 20; 35%). Non-statistically-significant associations were observed between the presence of *T. tenax* and age, gender, smoking habit or diabetes. Statistically significant associations were observed between the presence of *T. tenax* and periodontal disease, and between *T. tenax* and the Periodontal Screening and Recording (PSR) index.

**Conclusion:**

*T. tenax* showed a high presence in patients with progressive states of periodontal diseases. Consequently, *T. tenax* detection is strongly recommended in patients with periodontal disease diagnosis and with a PSR index greater than 3.

**Electronic supplementary material:**

The online version of this article (10.1186/s12903-019-0885-3) contains supplementary material, which is available to authorized users.

## Background

*Trichomonas tenax* is a flagellated, aerotolerant protozoan that lives in the human oral cavity and is distributed between the teeth, gums, tongue and saliva of people with poor oral hygiene [1]. The presence of this protozoan is considerably high in persons with more dental calculus, coated tongue and poorly-cleaned periodontal tissue rather than in individuals with clean and healthy oral cavities [2]. *T. tenax* has been implicated in the aetiology of several infections outside of the oral cavity, being detected in cerebrospinal fluid samples from patients diagnosed with polymicrobial meningitis [3], in the salivary duct infecting the sub-maxillary gland [4], and causing pulmonary eosinophilia in bronchoalveolar fluid [5]. *T. tenax* has also been identified in fibrocystic mastopathy [6], in a infra-auricular lymph nodes causing cervical adenopathy [7], in sputum samples of immunocompromised patients with chest pain and chronic lung diseases causing pulmonary trichomoniasis [8], and in pleural empyema samples from upper respiratory tract infections [9]. Although *T. tenax* has been detected in dental calculus and subgingival dental plaque, its role in the physiopathology and the mechanism involved on tissue damage of oral infections are still unclear [10]. In addition, *T. tenax* presence in the oral cavity has been associated with periodontal disease [11]; however, its role in this pathology is also unclear.

Periodontal disease occurs when the complex composition and organization of the periodontium is affected by a homeostatic interruption between the oral microbiome and the host, thus leading to the development of gingivitis and periodontitis, two related diseases that differ in their degree of periodontium commitment [12, 13]. Dental plaque-induced gingivitis (DPIG) is an inflammatory alteration of the soft tissue surrounding the teeth and gums, resulting from bacterial plaque accumulating on the teeth; it is clinically characterized by reddened and inflamed gums and increased gum bleeding after soft probing, which is reversible once bacterial plaque is eliminated by effective mechanical oral hygiene [14]. Gingivitis can also be modified by several factors, such as tobacco, drugs and hormonal changes occurring during puberty and pregnancy [15]. Periodontitis is considered as a progression of gingivitis, traditionally caused by bacterial plaque and characterized mostly by irreversible destruction of the supporting tissues around the teeth, periodontal ligaments, bone and soft tissue [16]. Clinically, during periodontitis, the epithelium migrates along the radicular surface, with insertion loss, increased pocket depth, and bone crest loss, which can lead to tooth loss [15, 16]. Therefore, periodontitis is the most severe and important kind of the periodontal diseases.

*T. tenax* presence in periodontal diseases has been reported since the 1960s, with research interest increasing from the 1980s to the present [11]. Thus, several reports about *T. tenax* prevalence support the idea that the parasite prevalence in oral infections was much greater in patients with periodontal diseases when compared with patients with periodontal oral health [2, 11]. In most such reports, *T. tenax* was detected from bacterial plaque samples by direct microscopy or culture, with prevalences that varied significantly (0–94%) [11]. The wide range in estimated prevalences likely reflect that *T. tenax* trophozoites are highly sensitive to environmental changes, such as temperature and pH affecting their shape and flagella size, which make the culture and maintenance of trophozoite morphology and viability, required for subsequent microscopic identification, more difficult [1]. Hence, detecting protozoan by a more sensitive technique, such as polymerase chain reaction (PCR), could greatly improve the estimation of *T. tenax* prevalence in patients with periodontal disease. However, from our understanding only four prevalence studies have used PCR to detect this protozoan [17–20]. In Chile, the knowledge of *T. tenax* prevalence in patients with periodontal disease is poorly known, with a single report on prevalence from 1978, which showed a prevalence 38%, using microscopic detection of *T. tenax* trophozoites for their identification [21].

In the present study, PCR was used to evaluate the presence of *T. tenax* in patients with periodontal disease, in order to update the prevalence data for this protozoan and to analyse its association in the development of periodontal disease and the presence of risk factors for establishing the parasite infection.

## Methods

### Patients and samples

We performed an observational, and cross-sectional study from May 2013 to November 2014, on people ranging from 20 to 80 years old, who were attended at the Periodontics Post-Graduate Clinic of the University of Antofagasta, Antofagasta, Chile. Inclusion criteria were the clinical diagnosis of periodontal disease. Exclusion criteria for patients were the followed: 1) not having received periodontal treatment during the last year, 2) not having taken antibiotics treatment during the last 6 months, and 3) not having dental implants. Following this, 50 patients were selected based on their periodontal diagnosis and they were classified initially according to the criteria of the American Academy of Periodontology (AAP) [22], and re-classified according to the recently guidelines to classification of periodontal disease [23] into dental plaque-induced gingivitis (DPIG) or periodontitis. Thirty patients had generalized periodontitis, where stage ranged from I to III, and grade ranged from A to B; and 20 patients had DPIG. For both groups, dental plaque and dental calculus samples were taken prior to the initiation of periodontal therapy. All patients included in the study gave their informed consent, which was approved by the Research Ethics Committee of the University of Antofagasta (CEIC-REV/2014).

Samples of dental plaque and dental calculus from patients with periodontitis were obtained from the periodontal pocket with a probing depth ≥ 5 mm. Dental plaque samples were obtained through a gutta-percha cone, previously disinfected with 0.12% chlorhexidine, which was introduced subgingivally and rubbed for 5 s on the contaminated tooth surface; meanwhile, the dental calculus samples were obtained using a sterile curette. Both samples from each patient were deposited and dispersed into an independent microtube containing 1.5 mL sterile Ringer’s solution. Moreover, samples of dental plaque and dental calculus from patients with DPIG were obtained with a sterile curette, deposited and dispersed into a microtube containing 1.5 mL of sterile Ringer’s solution. All samples were transported to the Molecular Parasitology Laboratory at the University of Antofagasta within 2 h of collection and immediately processed to maintain protozoan viability to avoid the trophozoites lysis.

### Clinical registry data

Demographic antecedents, such as gender and age, and risk factors such as diabetes status and smoking habits, were registered from clinical records of patients diagnosed with periodontitis or DPIG. Furthermore, the Periodontal Screening and Recording (PSR) index was recorded, which consists of an examination of six sites per tooth for all the patient’s teeth, where the end of a periodontal WHO probe [24] was inserted gently between the tooth and gum to the depth of the dental groove. The probing depth was read by observing the position of the black band on the probe, granting each sextant a PSR index (between 0 to 4) and registering the highest code per sextant [25]. Similarly, the Gingival Index (GI) was recorded, following the classification criteria (between 0 and 3) described by Löe et al. [26]. Also, clinical images of each patient’s teeth were recorded to document the periodontal status.

### Direct observation

All samples in Ringer’s solution were centrifuged at 800 x g for 10 min at 20–22 °C. The supernatant was discard and the pellet was resuspended in 500 μL of sterile PBS pH 7.2. Drops of each resuspended pellet were placed on microscope slides and examined under optic microscopy. The identification of *T. tenax* was established according to movement criteria (circular movement) under dry 400x magnification, and according to morphological criteria (pear-shaped flagellated trophozoite, about 5–13 μm long) in samples stained with Giemsa and examined under 1000x immersion magnification. The remaining sample was used for DNA extraction.

### Genomic DNA extraction

Genomic DNA was extracted from dental plaque and/or dental calculus samples using the inorganic method of phenol-chloroform [27]. Briefly, sediment samples were re-centrifuged at 800 x g for 10 min at 20–22 °C, and the pellet was lysed with 500 μL of lysis buffer (50 mM Tris-HCl, pH 8.0, 4% Triton X-100, 62.5 mM EDTA pH 8.0, and 2.5 M LiCl) with 1 mg/mL of RNAse A (Thermo Fisher Scientific Inc., Waltham, MA, USA), incubated for 30 min at 37 °C, and then incubated for 2 h at 50 °C with 100 μg/mL of proteinase K (US Biological Life Sciences, Salem, MA, USA). Next, an equal volume of phenol-chloroform-isoamyl alcohol (25:24:1) was added. The suspension was vigorously stirred and centrifuged at 13,000 x g for 10 min at 4 °C. The aqueous phase was collected and transferred into a new tube, then an equal volume of chloroform was added and centrifuged at 13,000 x g for 10 min at 4 °C. The aqueous phase was recovered and transferred into a new tube. The DNA sample was precipitated with 0.3 M sodium acetate at pH 5.5 and a 2.5 volumes of cold absolute ethanol. The sample was centrifuged at 13,000 x g for 20 min at 4 °C. The supernatant was discarded, and the precipitate was washed with 500 μL of 70% ethanol, then centrifuged at 13,000 x g for 5 min at 4 °C. Finally, the precipitate was dried and resuspended in 50 μL of sterile free-nuclease water. Additionally, genomic DNA from *T. tenax* strain Hs-4:NIH (ATCC® 30207™) was extracted using the same method described.

### PCR amplification of the beta-tubulin gene

PCR was performed to detect the *T. tenax* beta-tubulin gene. The primers used were designed from the DNA sequence of the *Trichomonas vaginalis* beta-tubulin gene (accession number: XM_001582993) [28] to amplify a 405-bp product. The primer sequences were Tt β-tub (sense) 5′-ATACTCTATCGTCCCATCTC-3′ and Tt β-tub (antisense) 5′-GCCATCATGTTCTTGTTATCG-3′. The PCR reaction was performed in a reaction volume of 50 μL, containing: 1 μL of DNA, 1X *Taq* DNA buffer (75 mM Tris-HCl pH 8.8, 20 mM (NH_4_)_2_SO_4_, 0.01% (v/v) Tween 20), 0.2 mM of each dNTP, 2.5 mM of MgCl_2_, 400 nM of each primer, and 2.5 U of Taq DNA polymerase (Thermo Fisher Scientific, Inc., Waltham, MA, USA). The reaction was performed in a T100 thermocycler (Bio-Rad), using the following reaction conditions: denaturation at 95 °C for 3 min, followed by 35 cycles of denaturation at 95 °C for 30 s, primer annealing at 46.5 °C for 20 s, elongation at 72 °C for 1 min, and a final stage of 72 °C for 7 min. The amplification products were separated by electrophoresis in 1% agarose gel and stained with ethidium bromide (EtBr). Gel images were photo-documented using an MF-ChemiBIS 2.0 Gel Documentation System (DNR Bio-Imaging Systems, Mahale HaHamisha, Jerusalem, Israel) using Gel Capture Pro software.

### Statistical analysis

Chi-square analysis was used to verify the independence hypotheses in two variables and an alternative hypothesis of association between the variables. Also, the Fisher’s exact test was used for small samples. Binary logistic regression analysis was used to model the probability of detecting *T. tenax* presence based on the predictive variables, PSR and smoking habits. A statistical significance criterion level of *p* < 0.05 was used. All statistical analyses were performed using Minitab v.16.0 (Minitab, LLC, State College, PA, USA).

## Results

**Fig. 1:**
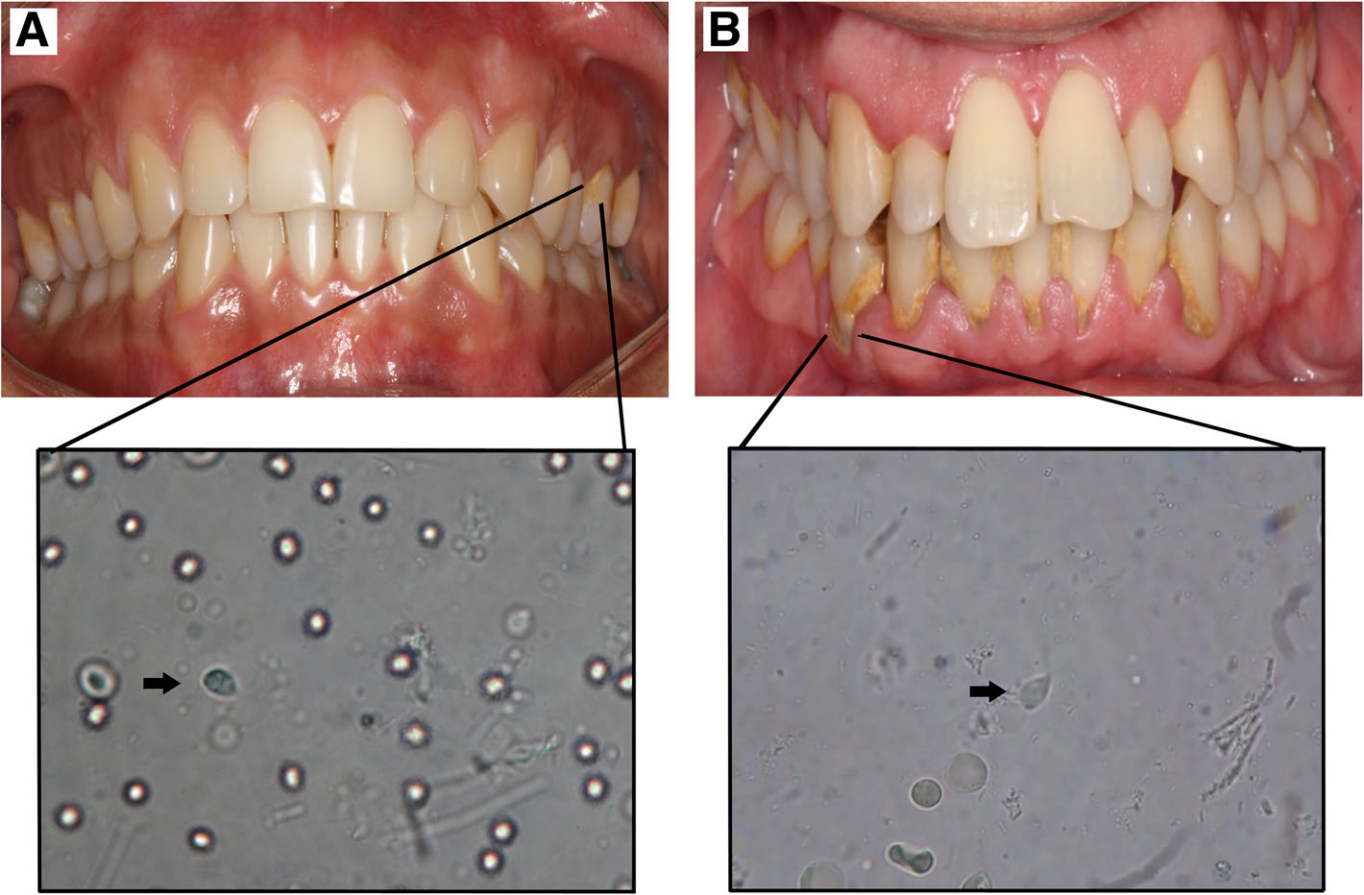
Direct microscopy observation of *T. tenax* in patients with periodontal disease. **a** Representative image from a dental plaque-induced gingivitis patient. **b** Representative image from a periodontitis patient. Bottom: *T. tenax* trophozoites observed by microscopy in each patient are showed by arrows (× 400)

*T. tenax* was observed by direct microscopy in patients with dental plaque-induced gingivitis and periodontitis (Fig. 1). Presence or absence of *T. tenax* in patients with periodontal disease was determined by the presence of a 405-bp PCR product for the β-tubulin gene in an agarose-gel electrophoresis (See Additional file 1: Figure S1 and Additional file 2: Figure S2). Amplification of the β-tubulin gene was also observed in samples from dental calculus and sub-gingival dental plaque from all those patients where *T. tenax* was recorded. From the 50 samples evaluated by PCR for *T. tenax* identification, 28 (56%) showed *T. tenax*, of which, 12 corresponded to female patients (24%) and 16 to male patients (32%), with no association between patient gender and *T. tenax* presence (χ^2^ = 2.131, *p* > 0.05) (Table 1). Examination of *T. tenax* presence by age group, showed a higher frequency of *T. tenax* in the 20-to 40-year-old group with 14 positive patients (50%), followed by the 40-to 60-year-old group with 11 positive patients (34%). No association was found between patient age and *T. tenax* presence (χ^2^ = 1.811, *p* > 0.05) (Table 1).

**Table 1:** *Trichomonas tenax* prevalence by demographic data

	Samples (n)	Presence of *T. tenax*		*p* value*
		YES n (%)	NO n (%)	
Gender				
Male	24	16 (67)	8 (33)	0.144
Female	26	12 (46)	14 (54)	
Total n (%)	50 (100)	28 (56)	22 (44)	
Age (years)				
20–40	29	14 (50)	15 (68)	0.404
40–60	17	6 (27)	11 (39)	
60–80	4	1 (5)	3 (11)	
Total n (%)	50 (100)	21 (42)	29 (58)	

* Chi-square test. Significantly different *p* < 0.05

*T. tenax* frequency by periodontal diagnosis in the study population (Table 2), showed that the protozoan was present in 7/20 patients (35%) with DPIG and in 21/30 patients (70%) with periodontitis, with a statistically significant association between *T. tenax* presence and periodontal diagnosis (χ^2^ = 5.965, *p* < 0.05). When analysing the association between smoking and *T. tenax* presence (Table 3), among the 28 patients with oral trichomoniasis, 13/28 (52%) were smokers and 15/28 (60%) were non-smokers, indicating no association between smoking habit and *T. tenax* presence (χ^2^ = 0.324, *p > 0.05*). *T. tenax* were only observed in 7/30 diabetic patients with periodontitis (23.3%), but not in diabetic patients with dental plaque-induced gingivitis 0/20 (0%). A statistically significant association between diabetes and periodontal diseases was observed (χ^2^ = 5.426, *p =* 0.02). The frequency of *T. tenax* in patients with periodontitis and diabetes was 6/7 (86%) (Table 3); however, no association between diabetes and *T. tenax* presence was observed (Fisher’s exact test, *p* = 0.393). With regard to the gingival index and *T. tenax* infection, the range in which *T. tenax* was most frequently detected was the GI between 1.1 and 2, reported from 23/50 patients, representing 46% of the cases studied. No association was found between the GI and *T. tenax* presence (χ^2^ = 1.713, *p* < 0.05) (Table 3).

**Table 2:** *Trichomonas tenax* infection prevalence by periodontal diagnosis

Periodontal diagnosis	Samples (n)	Presence of *T. tenax*		*p* value*
		YES n (%)	NO n (%)	
Gingivitis	20	7 (35)	13 (65)	0.0145
Periodontitis	30	21 (70)	9 (30)	
Total n (%)	50 (100)	28 (56)	22 (44)	

* Chi-square test. Significantly different *p* < 0.05

**Table 3:** *Trichomonas tenax* prevalence by risk factors and gingival index

Variable	Samples (n)	Presence of *T. tenax*		*p* value*
		YES n (%)	NO n (%)	
Smoking habit				
YES	25	13 (52)	12 (48)	0.568
NO	25	15 (60)	10 (40)	
Total n (%)	50 (100)	28 (56)	22 (44)	
Diabetes mellitus^(a)^				
YES	7	6	1	0.300
NO	23	15	8	
Total n (%)	30 (100)	21 (70)	9 (30)	
Gingival index				
0.1–1	5	3	2	0.424
1.1–2	43	23	20	
2.1–3	2	2	0	
Total n (%)	50 (100)	28 (56)	22 (44)	

^a^ Fisher’s exact test =0.393. * Chi-square test. Significantly different *p* < 0.05

Finally, relative to *T. tenax* detection and the PSR index (Table 4), our results indicated that the range in which *T. tenax* was most frequently detected was in patients with PSR index between 3.1 and 4, with 19/23 patients in this range, representing 38% of the cases studied. This indicated an association between *T. tenax* presence and the PSR index (χ^2^ = 6.579; *p* < 0.05). Additionally, logistic regression analysis for the probability of detecting *T. tenax*, considering the predictive variables PSR index and smoking, showed no association between smoking habit and *T. tenax* presence (binary logistic regression *p* = 0.127). However, the probability of detecting *T. tenax* in non-smokers patients was greater than the probability of detecting the protozoan in smokers, at all PSR levels.

**Table 4:** *Trichomonas tenax* infection prevalence by Periodontal Screening and Recording (PSR) index

Periodontal Screening and Recording index	Samples (n)	Presence of *T. tenax*		*p* value*
		YES n (%)	NO n (%)	
1.1–2	12	5 (42)	7 (58)	0.0372
2.1–3	12	4 (33)	8 (27)	
3.1–4	26	19 (73)	7 (27)	
Total n (%)	50 (100)	28 (56)	22 (44)	

* Chi-square test. Significantly different *p* < 0.05

## Discussion

For many years, it was assumed that bacteria were the only microorganisms involved in the formation of dental plaque, and they were also considered responsible for forming the dental calculus [29]. However, although largely comprised of bacteria, some fungal, mycoplasma and protozoan species are also found in dental plaque and calculus, among these the flagellated protozoan, *Trichomonas tenax* [11, 30]*.*

Most efforts to understand the oral microbiome have focused on the bacteriological microbiota, while oral parasitology has been less studied [31]. *T. tenax* has recently been reported to produced damage to the mammalian epithelial cells, and it behaves similarly to *T. vaginalis*, a closely related and pathogenic *Trichomonas* species of the genitourinary tract, thus satisfying the requirements to be considered as a parasite [32]. Therefore, the traditional view of *T. tenax* as a commensal organism is now being questioned [32]. However, *T. tenax’s* pathophysiological role in periodontal disease is unclear, since no studies in animal models have corroborated its pathological nature [11]. For these reasons, accurate identification of *T. tenax* in patients with periodontal disease is required, in order to update our understanding of prevalence and determine its association with the disease.

Currently the *T. tenax* prevalence range in patients with periodontal disease is very wide (between 0 to 94%). This likely reflects the use of different detection methodologies, mainly comprised of conventional detection methods such as microscopic observation and cell culture, which require great operator skill and meticulous control of the conditions that allow trophozoite viability [11]. This problem could be solved by using a more sensitive and specific techniques for identification, such as PCR, which is already used to identify bacterial microorganisms in the oral cavity associated with periodontal disease [33, 34]. In this study, we used PCR with primers designed to amplify a segment of 405 bp of the beta-tubulin gene. This allows the specific amplification of amounts as low as 100 fg of *T. tenax* DNA, without interference from DNA belonging to other known pathogens of the oral cavity [35], such as *Porphyromonas gingivalis*, a well-known periodontal pathogen that is frequently found in patients with chronic periodontitis [29], or *Aggregatibacter actinomycetemcomitans*, whose colonization has been associated with aggressive periodontal disease [36]. The frequency of *T. tenax* detected by PCR in patients with periodontal disease was in agreement with previous studies using PCR to identify *T. tenax*, where the prevalence observed ranged between of 6 to 56% [17, 19, 20, 37]. A difference was also observed in *T. tenax* presence relative to periodontal disease severity (progression from dental plaque-induced gingivitis to periodontitis), being more frequently recorded in patients with periodontitis rather than in patients with gingivitis. This is likely because periodontitis involves destruction of the insertion periodontium, generating epithelial migration along the radicular surface and increasing the periodontal pocket depth, which generates anaerobic conditions that favour the establishment of the protozoan [15]. This could explain why some authors have reported higher *T. tenax* prevalence in patients with periodontitis rather than in patients with gingivitis [18, 38, 39]. However, no such differences have been reported in other studies where detection methods such as microscopy or cell culture were used [40, 41]. We demonstrated a statistically significant association between *T. tenax* presence and periodontal diagnosis: as such, screening for this protozoan needs to be considered in patients diagnosed with periodontitis.

In addition, the observational study carried on demonstrated for the first time the association between *T. tenax* presence and the PSR index. This index is based on three parameters: gingival bleeding on probing, calculus accumulation, and depth of probing, providing a detailed view of the patient’s periodontal status [42]. The association between *T. tenax* presence in patients with PSR indexes ≥3 allows us to consider the importance of this parameter in the screening for *T. tenax* infection in patients with periodontal disease. This observation is supported as deeper periodontal probing means that the periodontal environment becomes more anaerobic. This leads to a decrease in the partial pressure of oxygen, potentially explaining why the periodontal pocket depth is a critical factor for the colonization and anaerobic growth of *T. tenax* [11]. This observation was supported by our positive results for the presence of *T. tenax* in patients with PSR index of 3 or 4 and probing at depths greater than 5 mm.

In Chile, only a single previous study examining the prevalence of *T. tenax* exists, conducted in the city of Valdivia [21]. Here, the authors used identification by optical microscopy, a reported 38% frequency (*n* = 50) of patients infected with *T. tenax* [21]. This frequency was lower than that reported here (56%), likely partly because our patients had not been examined or clinically treated before, and that the authors’ methodology (direct observation) was less sensitive for detecting protozoa compared to PCR.

No statistically significant association was found between gender and *T. tenax* presence. These results are in agreement with those obtained in other studies [39, 43]. Some studies have reported that *T. tenax* detection is influenced by age, where its prevalence is higher in adolescents than in children [44]. It has also been reported that periodontal tissues in patients over 40 years old have greater *T. tenax* infections [45, 46]. However, our study did not find evidence of a significant association between age and *T. tenax* infection. Recently, a review of *T. tenax* prevalence in periodontal diseases showed that among eight studies focused on groups of children and students, the prevalence was quite low, ranging from 0 to 4%, except in one study reporting a *T. tenax* prevalence of approximately 14% in young people [11]. Some authors have supported the idea that when age increases, *T. tenax* presence also increases [47, 48]. However, these reports are questionable since *T. tenax* presence in the oral cavity is closely linked to the presence of teeth, such as been reported in completely edentulous patients [49] or in very young children [44], where no *T. tenax* were detected.

With regard to risk factors for periodontal disease, no association between *T. tenax* presence and smoking was observed. This was in accordance with previously studies, where smoking was not associated with the presence of *T. tenax,* and smoking and non-smoking groups showed similar frequency of the protozoan [50]. However, our data for the probability of detecting *T. tenax* in non-smokers patients was greater than that for detecting the protozoa in smokers. That may be explaining because one of the first alterations in the periodontal tissues of smoking patients corresponds to epithelial hyperplasia and gingival recession [51], which are associated with vasoconstriction [52] and fewer blood vessels [53]. This causes less iron availability in the environment, which could affect *T. tenax* adherence to the gingival tissues, since studies of cytoadherence performed in the closely related species, *T. vaginalis,* indicate that adhesion levels are mediated specifically by iron [54]. Therefore, non-smokers could provide a more favourable environment for the development of the protozoan. Thus, further studies are needed with more smoking/non-smoking patients to corroborate the above results at experimental levels. However, the fact that smokers show reduced infection by *T. tenax* could suggest a protective effect of nicotine against infection by the parasite. Experimental studies have shown that nicotine has protective effect against pneumonia caused by *Pneumocystis carinii* [55]. Also, the periodontal pathogen *Porphyromonas gingivalis* can adapt to nicotine exposure over time and develops tolerance to the inhibitory effect of nicotine over proliferation [56].

Finally, diabetes mellitus (DM) is considered the major risk factor for development of periodontitis [57]. In these patients, diabetes could alter the local environment within the periodontal pocket, favouring the growth of certain oral pathogens [57]. The lack of association between diabetes and the presence of *T. tenax*, could be influenced by our low sample size for diabetic patients with periodontal disease, so we suggest that subsequent case/control studies are conducted at a population level for both variables. However, our data agree with others, since non-significant association between type 2 DM in patients with periodontitis and presence of oral pathogens, such as *A. actinomycetemcomitans*, *P. gingivalis* and *Fusarium nucleatum*, has been reported [58]; and also, non-association has been showed between diabetes and presence of *T. tenax*. [59]

## Conclusion

We used PCR to detect *T. tenax* and revealed that the protozoan was frequently present in patients with periodontal disease, with an increased frequency of *T. tenax* in patients with periodontitis rather than in dental plaque-induced gingivitis. In addition, we demonstrated an association between periodontal disease development and *T. tenax* presence, and for first time demonstrated an association between the PSR index and *T. tenax* presence. Thus, we recommend screening for this protozoan in patients with periodontal disease and higher PSR indexes (between 3 and 4), mainly due to the risk of infection in other locations outside the oral cavity.

## Additional files

Additional file 1: **Figure S1.** PCR of β-tubulin gene for *T. tenax* detection in patients diagnosed with periodontitis. β-tubulin PCR products of periodontitis patients (samples 1 to 30) were separated by electrophoresis in 1% agarose gel and stained with Ethidium Bromide (EtBr). M: 100-bp molecular ladder marker. Pos: *T. tenax* strain Hs-4:NIH genomic DNA. Neg: water. (TIF 8004 kb)

Additional file 2: **Figure S2.** PCR of β-tubulin gene for *T. tenax* detection in patients with dental plaque-induced gingivitis. β-tubulin PCR products of gingivitis patients (samples 1 to 20) were separated by electrophoresis in 1% agarose gel and stained with Ethidium Bromide (EtBr). M: 100-bp molecular ladder marker. Pos: *T. tenax* strain Hs-4:NIH genomic DNA. Neg: water. (TIF 7791 kb)

## Data Availability

The data analysed during this study are included in this published paper and Additional file 1. The dataset used is available from the corresponding author on reasonable request.

## Abbreviations

AAP: American Academy of Periodontology
DM: Diabetes mellitus
DPIG: Dental plaque-induced gingivitis
EtBr: Ethidium bromide
GI: Gingival index
PCR: Polymerase chain reaction
PSR: Periodontal Screening and Recording index
WHO: World Health Organization
