# Supplementary figures and images for: Observational cross-sectional study of Trichomonas tenax in patients with periodontal disease attending a Chilean university dental clinic
Source: BMC Oral Health. 2019 Sep 4;19:207. doi: 10.1186/s12903-019-0885-3 (PMC6727549; doi:10.1186/s12903-019-0885-3)

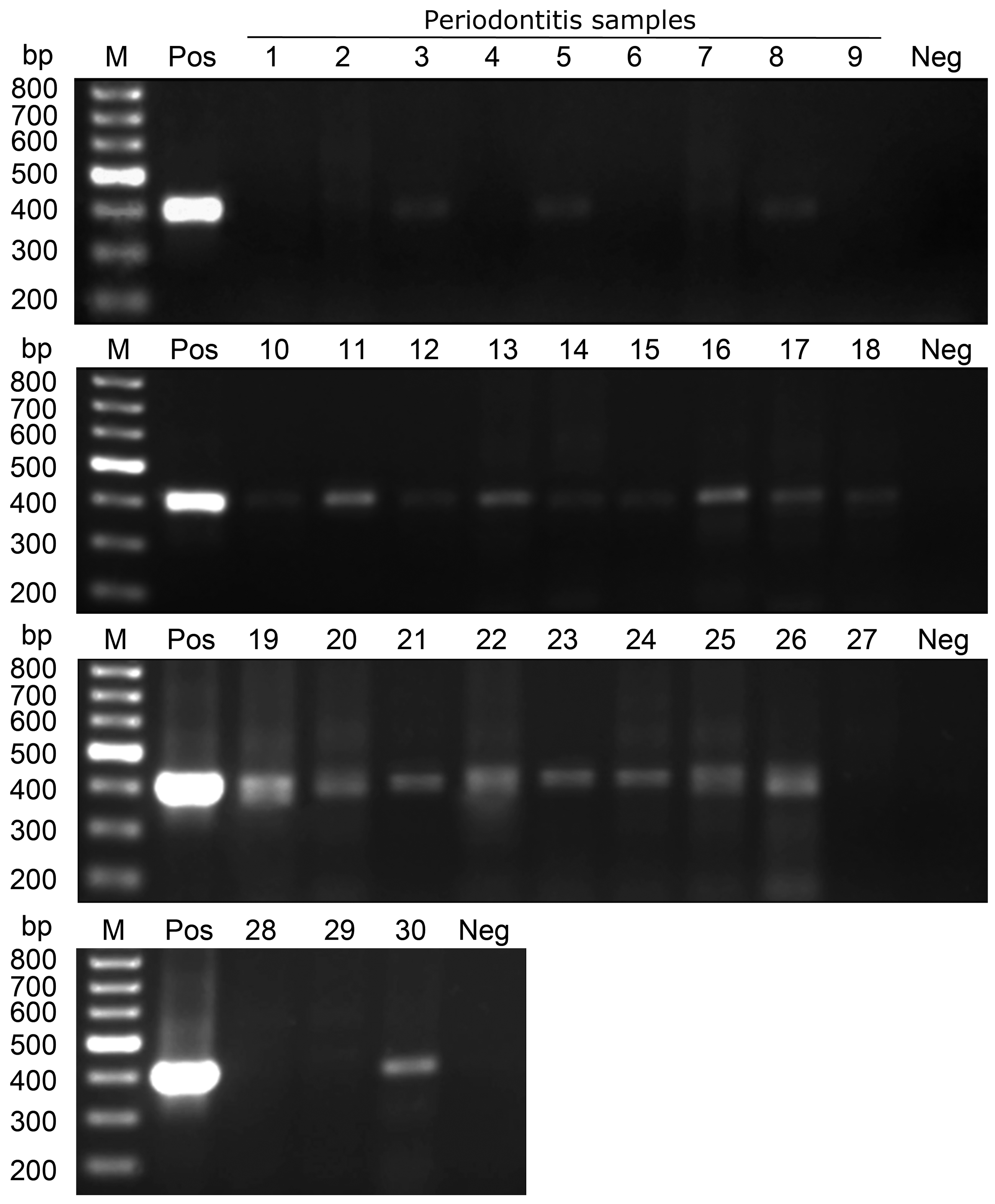

Supplement: Supplementary file 1 — Figure S1. PCR of β-tubulin gene for T. tenax detection in patients diagnosed with periodontitis. β-tubulin PCR products of periodontitis patients (samples 1 to 30) were separated by electrophoresis in 1% agarose gel and stained with Ethidium Bromide (EtBr). M: 100-bp molecular ladder marker. Pos: T. tenax strain Hs-4:NIH genomic DNA. Neg: water. (TIF 8004 kb) [file 12903_2019_885_MOESM1_ESM.tif]

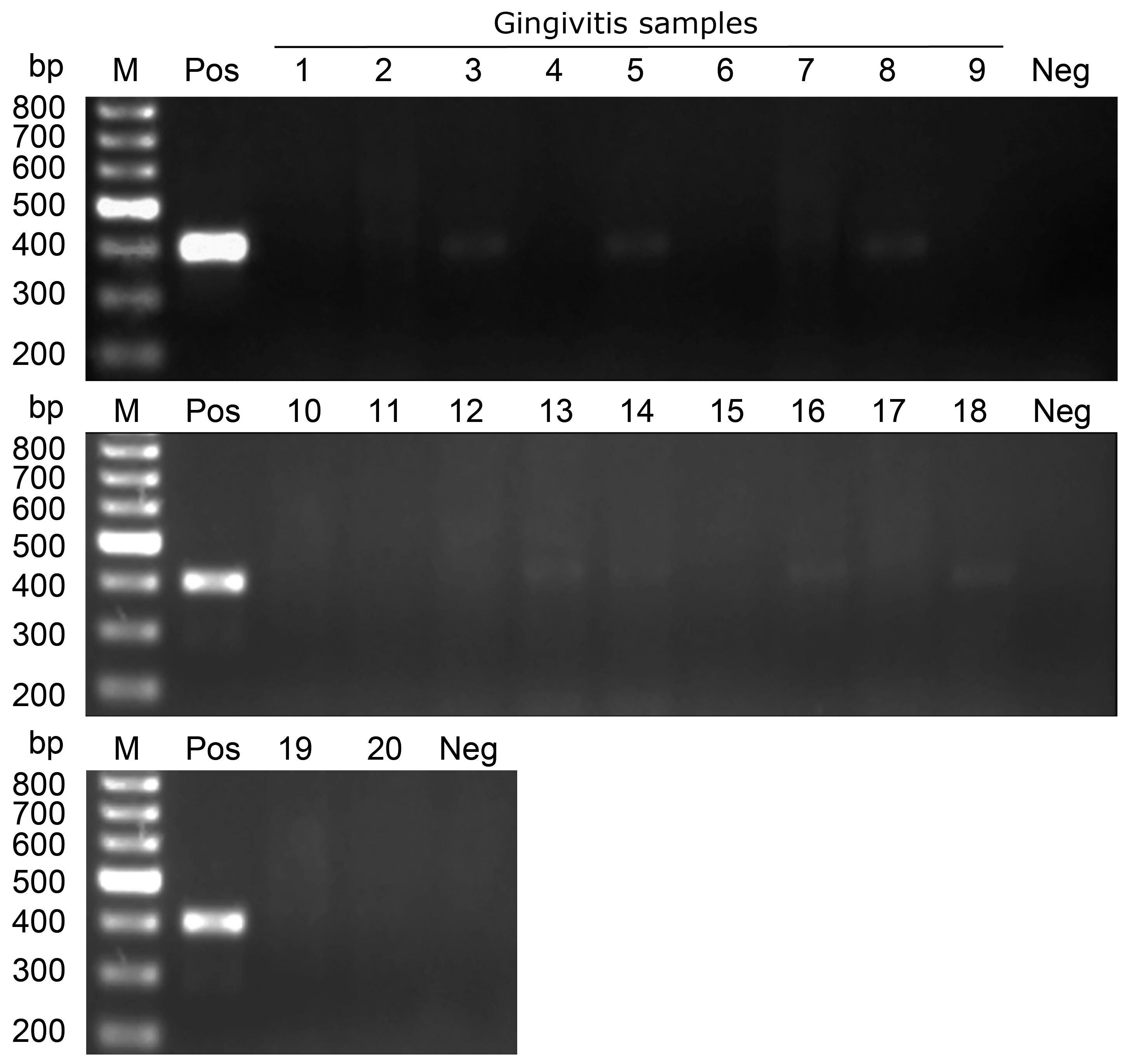

Supplement: Supplementary file 2 — Figure S2. PCR of β-tubulin gene for T. tenax detection in patients with dental plaque-induced gingivitis. β-tubulin PCR products of gingivitis patients (samples 1 to 20) were separated by electrophoresis in 1% agarose gel and stained with Ethidium Bromide (EtBr). M: 100-bp molecular ladder marker. Pos: T. tenax strain Hs-4:NIH genomic DNA. Neg: water. (TIF 7791 kb) [file 12903_2019_885_MOESM2_ESM.tif]
